# Comparative transcriptomic signatures of virulent and attenuated *Mycobacterium bovis* growing *in vitro* and in mice

**DOI:** 10.3389/fcimb.2025.1643664

**Published:** 2025-10-28

**Authors:** Hazem F. M. Abdelaal, Lama M. Salem, Howard Steinberg, Adel M. Talaat

**Affiliations:** ^1^ Department of Pathobiological Sciences, University of Wisconsin-Madison, Madison, WI, United States; ^2^ Computing Bioinformatic program, Faculty of Computers and Information, Menoufia University, Shebeen El-Kom, Egypt; ^3^ Vireo Vaccine Intl., Middleton, WI, United States

**Keywords:** *Mycobacterium bovis*, bacterial pathogenesis, transcriptomics, comparative genomics, BCG

## Abstract

Bovine tuberculosis (bTB), caused by *Mycobacterium bovis* (*M. bovis*), poses a significant global health and economic burden. Despite extensive research, a comprehensive understanding of *M. bovis* pathogenesis, particularly its transcriptional adaptation across different growth phases and within the host environment, remains incomplete. Here, we performed a comprehensive transcriptomic analysis of virulent *M. bovis* and the attenuated *M. bovis* BCG strain (BCG) across early-log, mid-log, and stationary growth phases to elucidate the molecular underpinnings of their phenotypic distinctions. Differential expression was computed with DESeq2, and coexpression modules were derived with WGCNA. Gene sets emphasized secretion systems and lipid metabolism. For biological context, selected transcripts were quantified by qRT PCR from lungs of infected C3HeB FeJ mice at four and sixteen weeks. Both strains remodeled transcription across growth, highlighting significant differences in pathways related to cell wall biosynthesis, lipid metabolism, transcriptional regulation, protein secretion, and the PE/PPE protein family. Notably, the Virulent *M. bovis* showed higher expression of envelope lipid genes, including the Pks13 and FadD32 locus, and a subset of DosR targets, while BCG emphasized stress and metabolic adjustment. Coexpression analysis provided a systems-level view of the transcriptional programs governing *M. bovis* and *M. bovis* BCG physiology, identifying key modules of co-expressed genes that regulate small molecules transport, amino acid biosynthesis and immune evasion in *M. bovis*. Furthermore, we analyzed *M. bovis* transcriptional responses during murine lung infection, identifying a core set of DEGs linked to host-pathogen interactions and mechanisms of persistence. These findings offer novel insights into *M. bovis* adaptation strategies and transcriptomic signatures that separate virulent *M. bovis* from attenuated BCG across growth and in the host. Differences in secretion capacity and lipid metabolism align with known deletions and attenuation mechanisms, and the *in vivo* measurements provide context for prioritizing pathways and BCG substrain evaluation.

## Introduction

Bovine tuberculosis (bTB), caused by *Mycobacterium bovis* (*M. bovis*), is a major animal and zoonotic disease prevalent in many countries, imposing substantial economic losses and posing a public health threat (Cosivi et al., 1998). In developing countries, where approximately 85% of global cattle and 82% of the human population reside, bTB often remains an underdiagnosed and inadequately controlled problem (Ayele et al., 2004). The presence of bTB undermines the development of the dairy and beef industries and acts as an impediment to international trade. Genomic comparisons reveal over 99.9% nucleotide sequence similarity between *M. bovis* and *Mycobacterium tuberculosis* (*M. tb*), the primary causative agent of human tuberculosis (Brosch et al., 2002; Smith et al., 2006). However, *M. bovis* exhibits distinct biological properties compared to *M. tb*, including differences in transmissibility, host range, antigenic composition, and virulence (Golby et al., 2007; Golby et al., 2013). A thorough understanding of the unique virulence mechanisms of *M. bovis* is crucial for developing novel and effective control strategies for bTB. Earlier studies aiming to decipher these virulence mechanisms, often comparing *M. bovis* to the attenuated *M. bovis* BCG vaccine strain, utilized DNA microarrays. While these identified some differentially expressed genes (DEGs) that were further analyzed in murine macrophages (Blanco et al., 2009), they lacked a comprehensive analysis of gene regulatory networks across different growth phases. Using a high throughput approach for transcriptional analysis, we employed RNA sequencing (RNASeq) to fill this knowledge gap and compare the transcriptome of virulent and vaccine strains of *M. bovis* during different phases of growth. Additionally, among the diverse BCG sub-strains, BCG Russia is categorized as an early strain and retains certain ancestral features, which may influence transcriptional behavior and virulence-associated pathways compared to late sub-strains such as BCG Pasteur (Elton et al., 2023).

Early on, several RNA-Seq approaches were employed to decipher gene regulatory network on a whole transcriptome level for members of *M. tb* complex (Chiner-Oms et al., 2019; Abdelaal et al., 2022). However, much of the analysis of bTB has concentrated on the host’s immune response to infection (Nalpas et al., 2013; McLoughlin et al., 2014; Abdelaal et al., 2022), with less attention paid to the *M. bovis* expressed genes during infection or across distinct physiological states. In cattle, RNA-Seq identified key transcriptional markers of infection when *M. bovis*-infected cows were analyzed, identifying genes such as GMBZ and CCL8 as significantly regulated genes during transition from early to chronic phases of infection (Abdelaal et al., 2022). Other studies identified genes associated with IL-17A expression, as indicators for the development of immune responses to *M. bovis* infection or immunization with effective vaccines directed against *M. bovis* (Waters et al., 2016). Unfortunately, comprehensive analysis of host-pathogen interactions during infection and on a genome-wide level, is difficult to conduct in the target host (e.g. cattle for *M. bovis*) or even animal models that lack key aspects of the disease. Recently, the C3HeB/FeJ mouse model which develops caseous necrotic granulomas; a hallmark of human and bovine tuberculosis lung lesions; was utilized to examine the molecular pathogenesis of *M. tb* and *M. bovis* infections (Kramnik and Beamer, 2016; Boute et al., 2017). This model was considered a significant advancement over traditional murine models (e.g BALB/c and C57Bl/6 mice) that lack granuloma structures observed during the target host infection (Calderon et al., 2013). Fortunately, necrotizing granuloma were observed in *M. bovis* infected C3H3B/FeJ mice associated with high bacterial and neutrophile loads (Boute et al., 2017), very similar to lungs of naturally infected cattle. This model was used in this study to examine the transcription of a selected list of genes with a unique *in vitro* transcriptional profile.

In this report, we first compared the gene expression profiles of virulent *M. bovis* and its attenuated BCG counterpart during *in vitro* growth, aiming to identify key genes crucial for the transitions between early-log, mid-log, and stationary growth phases. These genes are potentially significant contributors to the differential growth of each *M. bovis* strain. We found that the expression of genes encoding a range of functional activities varied significantly between the strains. Subsequently, we investigated the biological implications of these findings by analyzing the *M. bovis* transcriptome within the lungs of infected C3HeB/FeJ mice during both active and chronic phases of bTB. This approach allowed us to identify both unique and common transcriptional signatures of *M. bovis* during transition to different growth phases, with several genes found to be regulated both *in vitro* and during murine infection.

## Materials and methods

### Bacterial strains and media

*M. bovis* AF2122/97 and *M. bovis* BCG-Russia (BCG-1) strains were grown in at 37 °C in Middlebrook 7H9 broth (Difco) containing 10% albumin- dextrose-catalase (ADC), 0.5% Pyruvate, and 0.05% Tween 80. To define the distinct *in vitro* growth phases, we generated standard growth curves for both *M. bovis* and BCG Russia by measuring CFU/mL over time at 24-hour intervals (**Supplementary Figures S1A, B**). This allowed consistent and biologically validated sampling at early-log, mid-log, and stationary phase.

For RNA preparation, bacterial cultures at OD600 0.5, 1 and 2 were snap-frozen on ice and centrifuged at 3,000 × g for 10 min at 4°C. Bacterial pellets were then stored at -80°C. Bacterial stocks for mouse infections were prepared as previously described (AL et al., 1997).

### Mouse infections

C3HeB/FeJ mice groups (N = 20/each) at 5–6 weeks age was infected with approximately 100 CFU per mouse were administered by aerosol using the Glas-Col inhalation system (Glas-Col, LLC, Terre Haute, IN) as outlined before (Abdelaal et al., 2019). The infectious dose for each group was confirmed by plating lungs of an infected mouse at 1-day post- challenge. Mice were sacrificed at 4- and 16-weeks’ post infection for both histopathology bacterial CFU enumeration as detailed before (Marcus et al., 2016; Abdelaal et al., 2019).

### RNA extraction and preparation

RNA was isolated form frozen cultures using a TRIzol based method described previously (Abomoelak et al., 2009; Ward et al., 2010a; Ward et al., 2010b). RNA from murine lung tissues was isolated by homogenizing in TRIzol Reagent (Molecular Research Center, Cincinnati) and centrifuging at 3,000 × g for 5 min at 4 °C. Bacterial pellets were resuspended in 0.5 ml TRIzol Reagent containing 1% polyacryl carrier (Molecular Research Center), transferred to screw-caped tubes with 0.25 ml of zirconia/silica beads, and broken in a bead beater (Biospec Products, Bartlesville, OK). RNA was isolated by using TRIzol per the manufacturer’s instructions and treated with DNase I (DNA-free kit, Ambion). RNA was treated with TURBO DNase until PCR negative to remove contaminating DNA.

### RNAseq analysis

RNA-seq data analysis was performed on the CLC Genomics Workstation 8.0. Sequence reads were aligned to the *Mycobacterium bovis* AF2122/97 parental reference genome (GenBank accession number NC_002755). Raw sequencing reads underwent quality control using FastQC, and adapter trimming was performed with Trimmomatic to remove low-quality bases (Bolger et al., 2014). Reads were then aligned to the reference genome using HISAT2 (Kim et al., 2015). The reads per kilobase per million (RPKM) value for each gene was generated. Normalization was conducted using the median of ratios method within DESeq2 to account for sequencing depth and RNA composition biases (Love et al., 2014). Differential gene expression analysis using the R DEseq2 package was performed for the following groups of data sets for OD600 of 0.5, 1.0 and 2.0. The exact-test function was applied to determine the association of the differences in expression read counts within each group, and corresponding P-values were adjusted using the default Benjamini & Hochberg procedure (Benjamini et al., 2001; Reiner et al., 2003). Their adjusted P-values, in -log10 scale on the y axis and fold changes in log2 scale on the x axis, were plotted as a volcano plot. Differential gene expression was determined by a false discovery rate (FDR) threshold of p < 0.05. Genes with read counts of less than 5 were eliminated.

To identify gene co-expression modules associated with biological processes in *M. bovis*, we performed Weighted Gene Co-expression Network Analysis (WGCNA) using normalized gene expression data (Zhang and Horvath, 2005). Prior to network construction, lowly expressed genes were filtered, and variance-stabilized counts were used as input. A soft-thresholding power = 12 was selected using the scale-free topology criterion, ensuring optimal network fit and connectivity. A signed adjacency matrix was computed and transformed into a Topological Overlap Matrix (TOM), followed by hierarchical clustering to define co-expression modules. Gene module “eigengenes” were assigned distinct colors, and eigengenes were calculated to summarize module expression patterns. To investigate biological relevance, module-trait correlations were computed using sample metadata, identifying key modules associated with experimental conditions. Gene ontology (GO) enrichment and pathway analyses were performed using ClusterProfiler to assess functional relevance (Yu et al., 2012). Hub genes were determined based on intramodular connectivity, identifying core regulators within each module.

Inter-module relationships were visualized through eigengene expression analysis, highlighting shared and unique regulatory patterns. To enhance reproducibility, all analyses were performed in R using the DESeq2, WGCNA, and ClusterProfiler packages. This framework provided a robust systems-level approach for uncovering transcriptional signatures linked to growth-phase dynamics and virulence-associated differences in *M. bovis* strains.

### Quantitative real-time PCR analysis of mycobacterial transcripts

Primers with similar melting temperatures (60–66°C) were designed by using PRIMER 3 software (Rozen and Skaletsky, 2000). The sequences of primers are available on **Supplementary Table S1**. All primers were tested in PCRs with 100 AF2122/97 genome equivalents as template and the amplification products were evaluated by gel electrophoresis. A total of 1-2 μg RNA were used as template for cDNA using Superscript III. A SYBR green based qRT-PCR protocol utilizing GoTaq^®^ qPCR Master Mix (Promega, Madison, WI) and the Step One Plus TM Real-Time PCR System (Applied Biosystems^®^, Foster City, CA) were used. For *in vitro* samples, gene expression was normalized to 16S rRNA (rrs). For *in vivo* samples, expression was normalized to sigA (BQ2027_MB0760), a stable internal control during mycobacterial infection in mice. Analysis was carried out using LinRegPCR (Ruijter et al., 2009). Two biological replicates with no less than three technical replicates each were completed. Gene expression levels were determined using the ΔΔCt method (Livak and Schmittgen, 2001) for murine lungs relative to *in vitro* culture at mid-log phase. Statistical significance between groups was assessed using a one-way ANOVA with Tukey’s *post-hoc* test for multiple comparisons or an unpaired Student’s t-test for two-group comparisons.

### Statistical analysis section

Statistical analyses for RT-qPCR, bacterial enumeration, and growth curve assays were conducted using GraphPad Prism 9.4.1 (GraphPad Software, San Diego, CA, USA). Differences between groups were evaluated using a one-way analysis of variance (ANOVA) with Dunnett’s post-test to control for multiple comparisons. Growth curves were fitted using a nonlinear regression model to determine key growth parameters, including lag phase duration, maximum growth rate, and stationary phase plateau.

All statistical analyses were performed under parametric assumptions, with normality and homogeneity of variance assessed using the Shapiro-Wilk test and Levene’s test, respectively. A threshold of p < 0.05 was considered statistically significant. **Significant differences are labeled accordingly in the figures as * *p*  < 0.05, ** *p*  < 0.01, *** *p*  < 0.001, and **p < 0.0001.

## Results and discussion

### Characteristics of mycobacterial transcriptome

To profile *M. bovis* transcriptional machinery, we employed an RNA-Seq approach to identify genes associated with the growth of the virulent *M. bovis* AF2122/97 strain and the attenuated BCG-Russia strain. To capture the complete transcriptional landscape across different growth phases, cultures of *M. bovis* and *M. bovis* BCG (BCG) were harvested at early log (OD_600_ = 0.5), mid-log (OD_600_ = 1.0), and stationary phases (OD_600_ = 2.0) Growth curves for both strains are shown in **Supplementary Figure S1B**. This design enabled us to assess the dynamic transcriptional changes associated with bacterial growth at early-log, mid-log and stationary phases. The RNA-Seq analysis generated an average of 138 ± 5.2 million paired-end reads per library, exceeding previously defined quality control criteria for sequencing depth and alignment (Tarazona et al., 2011; Castel et al., 2015). On average, 91 ± 1.3% of reads mapped uniquely to the *M. bovis* AF2122/97 reference genome (GenBank accession number NC_002755). The RNA-Seq datasets detected transcripts for 64–66% of the coding regions in each sample, providing robust transcriptome coverage for 98.2% of the predicted genes encoded in *M. bovis* under all examined growth phases. The sample distance matrix showed clear clustering by biological replicate (**Supplementary Figure 2A**), and the library size distributions across samples (**Supplementary Figure 2B**). Together, these metrics ensured robust differential gene expression analysis. The summary statistics of the RNA-Seq data for each replicate are summarized in **Table 1**.

**Table 1:** Summary statistics for Illumina RNA sequencing data from individual samples.

Group/Replicate	(OD600)	Number of reads	Mapped reads in pairs	Mapped reads in broken pairs	% of mapped reads	% of mapped genes
Mbo-OD0.5-1	0.5	179,088,686	143,248,892	35,839,794	93.36	56.36764
Mbo-OD0.5-2	0.5	131,656,524	98,763,404	20,327,902	90.46	80.81154
Mbo-OD1-1	1	193,904,818	161,024,976	19,132,377	92.91	52.70105
Mbo-OD1-2	1	155,148,040	127,200,414	127,200,414	93.14	52.43217
Mbo-OD2-1	2	32,538,652	26,928,321	23,536,147	92.16	68.83403
Mbo-OD2-2	2	30,634,044	25,045,460	16,511,101	91.60	69.34735
BCG-OD0.5-1	0.5	164,360,570	129,471,154	21,038,933	91.57	96.6023
BCG-OD0.5-2	0.5	151,063,370	111,551,380	24,199,541	89.86	96.87118
BCG-OD1-1	1	150,539,890	109,999,798	27,153,812	91.11	96.62674
BCG-OD1-2	1	161,153,618	119,262,856	24,153,595	89.00	96.55341
BCG-OD2-1	2	153,514,454	120,747,084	19,050,340	91.07	96.65119
BCG-OD2-2	2	152,643,502	114,372,230	24,056,422	90.69	96.72452

### Differential gene expression across growth phases of *M. bovis* AF2122/97 and *M. bovis* BCG- Russia

**Figure 1:**
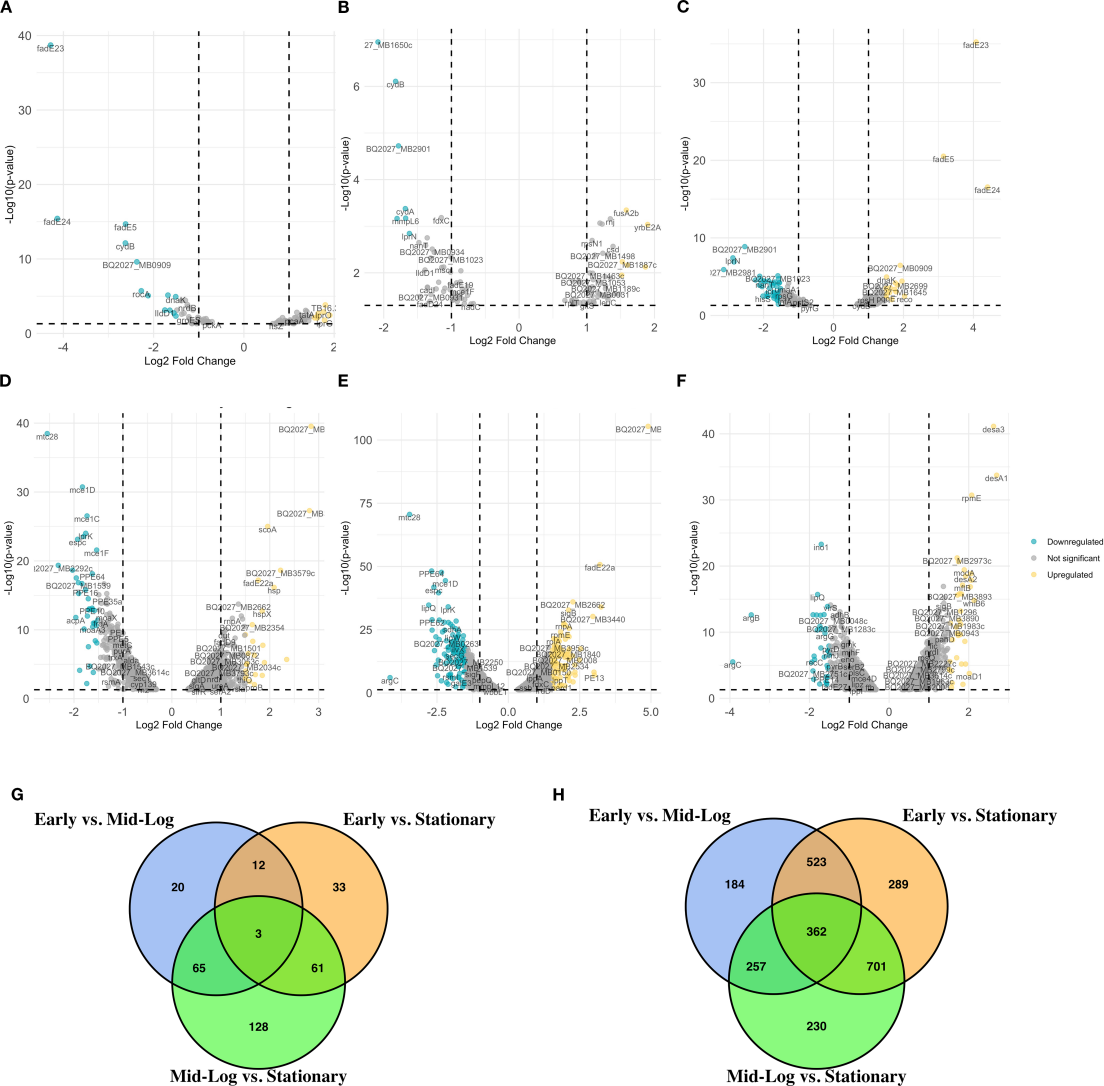
Differential gene expression analysis across growth phases of *M. bovis* and *M. bovis* BCG. Volcano plots represent the average log2 fold change versus the -log10 P-values between growth phases for *M. bovis* **(A–C)** and *M. bovis* BCG **(D–F)**. **(A)** Comparison of *M. bovis* OD600 0.5 vs OD600 1. **(B)** Comparison of *M. bovis* OD600 0.5 vs OD600 2. **(C)** Comparison of *M. bovis* OD600–1 vs OD600 2. **(D)** Comparison of *M. bovis* BCG OD600 0.5 vs OD600 1. **(E)** Comparison of *M. bovis* BCG OD600 0.5 vs OD600 2. **(F)** Comparison of *M. bovis* BCG OD600–1 vs OD600 2. **(G)** Venn diagram showing the overlap of significantly differentially expressed genes (DEGs) across the three pairwise growth phase comparisons in *M. bovis*, **(H)** Venn diagram showing the overlap of significantly DEGs across the three pairwise comparisons in *M. bovis* BCG. Teal dots represent downregulated transcripts, gold dots represent upregulated transcripts, and gray dots represent non-significant changes (fold change > ± 1 log2 and p < 0.05). Key differentially expressed genes are labeled. The differentially expressed genes [|log2FC|>1, FDR<0.05 (‘*’)] that are upregulated in *M. bovis* AF2122/97 or *M. bovis* AF2122/97-BCG-Russia.

To analyze the transcriptomic differences between the virulent and the attenuated bovine tubercle bacilli, we employed direct pairwise comparisons of the transcriptome of both organisms using a P-value threshold of < 0.05 and > ± 1.5-fold change. When early log (OD_600_ = 0.5) versus mid-log (OD_600_ = 1.0) cultures were compared in *M. bovis*, significant downregulation was observed for genes (n = 105) such as *fadE23, fadE24, and fadE5* (**Figure 1A**), essential components of lipid metabolism pathways (Crick and Guan, 2016), suggesting metabolic reprogramming during growth progression. Additionally, the downregulation of *cydB* and *ndh* highlights adjustments in respiratory pathways (Mittal et al., 2018). In contrast, BCG at the same transition exhibited significant upregulation of *BQ2027_MB1086* and *scoA* among the 126 upregulated and 95 downregulated genes (**Figure 1A**), pointing toward enhanced central metabolic activity in the attenuated strain (Houben et al., 2006).

When early log (OD_600_ = 0.5) versus stationary phase (OD_600_ = 2.0) cultures were compared in *M. bovis*, consistent downregulation of genes such as *BQ2027_MB1650c*, *cydB*, and *cydA* was observed, indicating shifts in respiratory and electron transport processes (**Figure 1B**) (Vaziri and Brosch, 2019). Upregulation of *yrbE2A, fusA2b*, and *rnj* suggests adaptive mechanisms involving nutrient acquisition and stress responses during exponential growth (**Figure 1B**) (Seshadri et al., 2009; Forrellad et al., 2013; Wang et al., 2022; Silva-Pereira et al., 2024). Similarly, BCG exhibited distinct metabolic changes with the upregulation of genes such as *fadE22a, BQ2027_MB2662*, and *PE12*, reflecting enhanced stress response mechanisms. Genes related to lipid metabolism, such as *mce1D* and *espc*, were significantly downregulated in BCG at this growth stage, consistent with its attenuated phenotype (**Figure 1E**) (Houben et al., 2006).

Finally, when comparing mid-log (OD_600_ = 1.0) versus stationary (OD_600_ = 2.0) phases, *M. bovis* exhibited upregulation of (n = 162) including dormancy- and survival-related genes, such as *fadE23, fadE24*, and *BQ2027_MB0909*, underscores its preparation for stationary phase and environmental adaptation (**Figure 1C**) (Houben et al., 2006). Concurrently, downregulation of genes (n = 189) such as *BQ2027_MB2981* and *BQ2027_MB1023* highlights the metabolic shifts favoring survival over replication (Vaziri and Brosch, 2019). In contrast, BCG demonstrated upregulation of stress response genes, such as *desa3, desA1*, and *modA* (**Figure 1F**) (Chang and Fox, 2006), while showing downregulation of genes like *ino1*, suggesting a diminished reliance on virulence-associated pathways (Movahedzadeh et al., 2004).

### Comparative transcriptomics of *M. bovis* and *M. bovis* BCG.

**Figure 2:**
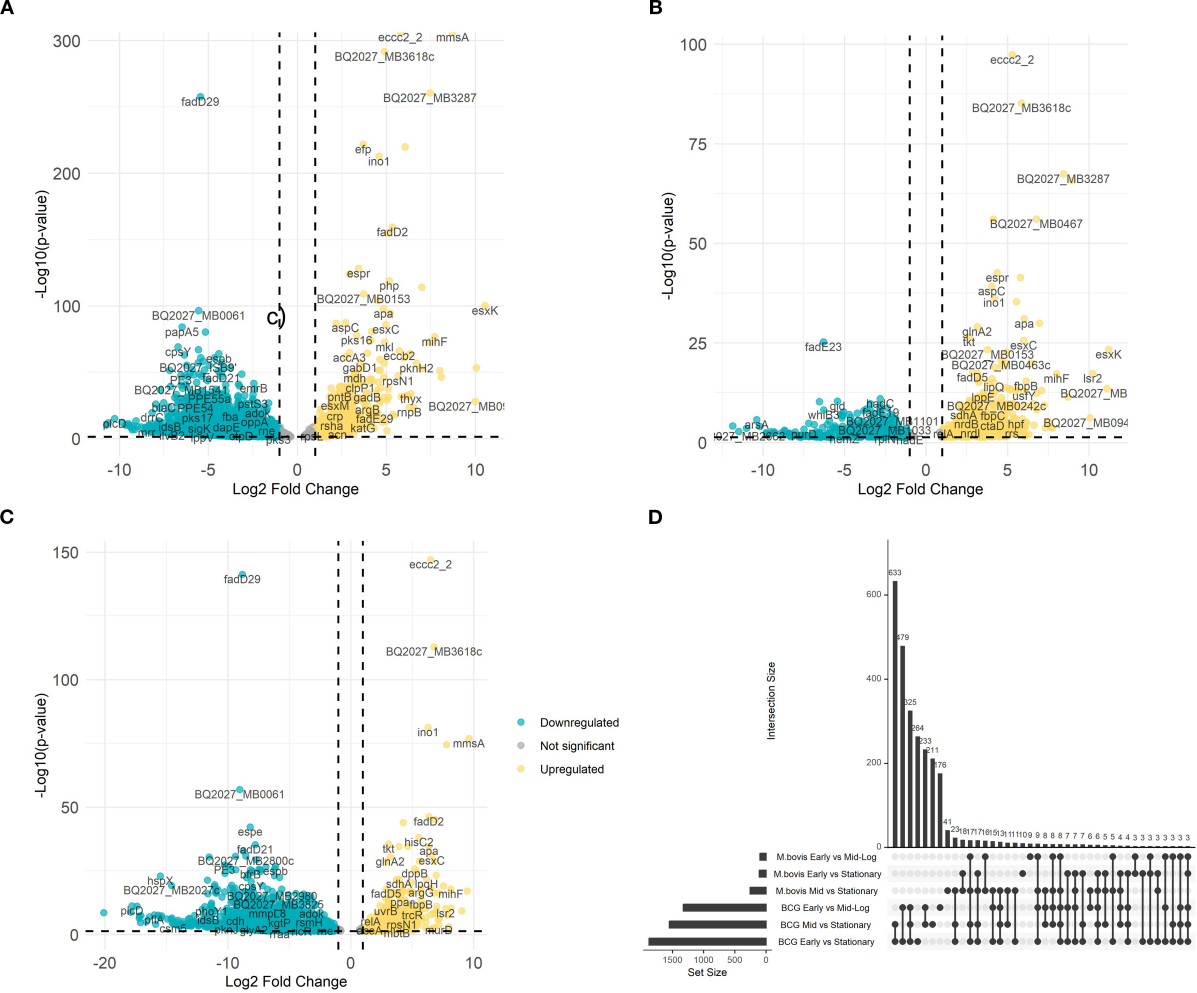
Comparative differential gene expression analysis between *M. bovis* and *M. bovis* BCG across growth phases. Volcano plots illustrate the average log2 fold change versus the -log10 P-values for *M. bovis* vs. *M. bovis* BCG at OD600 0.5 **(A)**, OD600 1.0 **(B)**, and OD600 2.0 **(C)**. Teal dots represent downregulated transcripts, gold dots represent upregulated transcripts, and gray dots represent non-significant changes (fold change > ± 1.5 log2 and p < 0.05). Labeled genes correspond to key differentially expressed genes. **(D)** UpSet plot depicting the overlap of differentially expressed genes among comparisons across early (OD600 0.5), mid-log (OD600 1.0), and stationary (OD600 2.0) phases for both *M. bovis* and *M. bovis* BCG. The differentially expressed genes [|log2FC|>1, FDR<0.05 (‘*’)] that are upregulated in *M. bovis* AF2122/97 or *M. bovis* AF2122/97-BCG-Russia.

To further explore the transcriptional divergence of *M. bovis* AF2122/97 and *M. bovis* BCG-Russia, we conducted direct pairwise comparisons between both strains growing at different growth phases. This analysis reveals phase-specific expression patterns that define the *M. bovis* virulent and attenuated strains (**Figure 2**).

At early growth phase, *M. bovis* significantly upregulates (n = 326) including key virulence-associated genes, such as *eccc2_2* (encoding ESX-II secretion-associated protein EccC2) and *mmsA* (encoding methylmalonate-semialdehyde dehydrogenase) (**Figure 2A**) (Gibson et al., 2022). The level of changes in gene expression ranged between log_2_FC = 5.79 to log_2_FC = 8.73 respectively. These Differentially Expressed Genes (DEGs) are integral to pathogenic mechanisms, including secretion systems and metabolic processes crucial for early-stage infection (Gibson et al., 2022). Although eccC2_2 expression was higher in M. bovis than in BCG, canonical ESX−1 genes (e.g., esxA/B, espA/C) were undetectable in BCG due to the stable deletion of the RD1 locus known to underlie its attenuation. The expression differences in eccC2_2 and mmsA do not themselves imply virulence roles but rather reflect strain-dependent variation in secretion-system activity and lipid metabolism.

At mid-log growth phase, the transcriptional landscape further differentiates (n = 374), with *M. bovis* continuing to show elevated expression of *mmsA* (MB0775c; methylmalonate−semialdehyde dehydrogenase), BQ2027_MB3287 (a putative metallopeptidase family protein), and BQ2027_MB0467 (exaC; NAD^+^−dependent acetaldehyde dehydrogenase), with log2 fold changes exceeding 6. Their regulation is consistent with known virulence strategies: metallopeptidases like Zmp1 are implicated in macrophage inflammasome suppression, and aldehyde dehydrogenation is required for detoxifying host-derived reactive aldehydes during persistent infection. Consequently, these genes are better framed as components of metabolic adaptation layers indirectly supportive of virulence (Guo et al., 2023). Meanwhile, *M. bovis* BCG demonstrates upregulation of stress-response and metabolic genes, indicative of its focus on environmental adaptability rather than virulence (**Figure 2B**).

At stationary phase, the virulent strain adapts to long-term survival, with pronounced upregulation of genes (n = 378) like *tkt* (encoding transketolase) and *apa* (encoding alanine-proline-rich antigen), highlighting a focus on dormancy and immune modulation (Sable et al., 2011; Fullam et al., 2012). In contrast, *M. bovis* BCG continues to prioritize stress response pathways, with limited expression of virulence-associated factors (**Figure 2C**). These findings illustrate the phase-specific transcriptional shifts underlying the divergent phenotypes of *M. bovis* and *M. bovis* BCG, providing insights into the molecular determinants of virulence and attenuation.

To further examine the overlap in differential gene expression across growth phases, we analyzed the intersection of differentially expressed genes between early, mid-log, and stationary phases for both *M. bovis* and *M. bovis* BCG (**Figure 2D**). The UpSet plot illustrates the extent of shared and unique transcriptional responses across growth transitions. Notably, *M. bovis* exhibits a greater number of overlapping DE genes between OD600 0.5 and OD600 1.0 (n = 31), suggesting a coordinated metabolic shift between early and mid-log phases. Conversely, *M. bovis* BCG shows fewer shared DE genes across phases, indicating a more gradual transcriptional adaptation with a focus on stress response mechanisms. The relatively high number of distinct genes (n = 42) differentially expressed only in the stationary phase in *M. bovis* further underscores its ability to enter a dormancy-like state, a feature less pronounced in *M. bovis* BCG. This comparative transcriptomic analysis highlights key regulatory mechanisms that distinguish virulence-driven metabolic shifts in *M. bovis* from the attenuation-associated transcriptional changes in BCG.

### Distinct modules underlying transcriptional changes during growth phase transition

**Figure 3:**
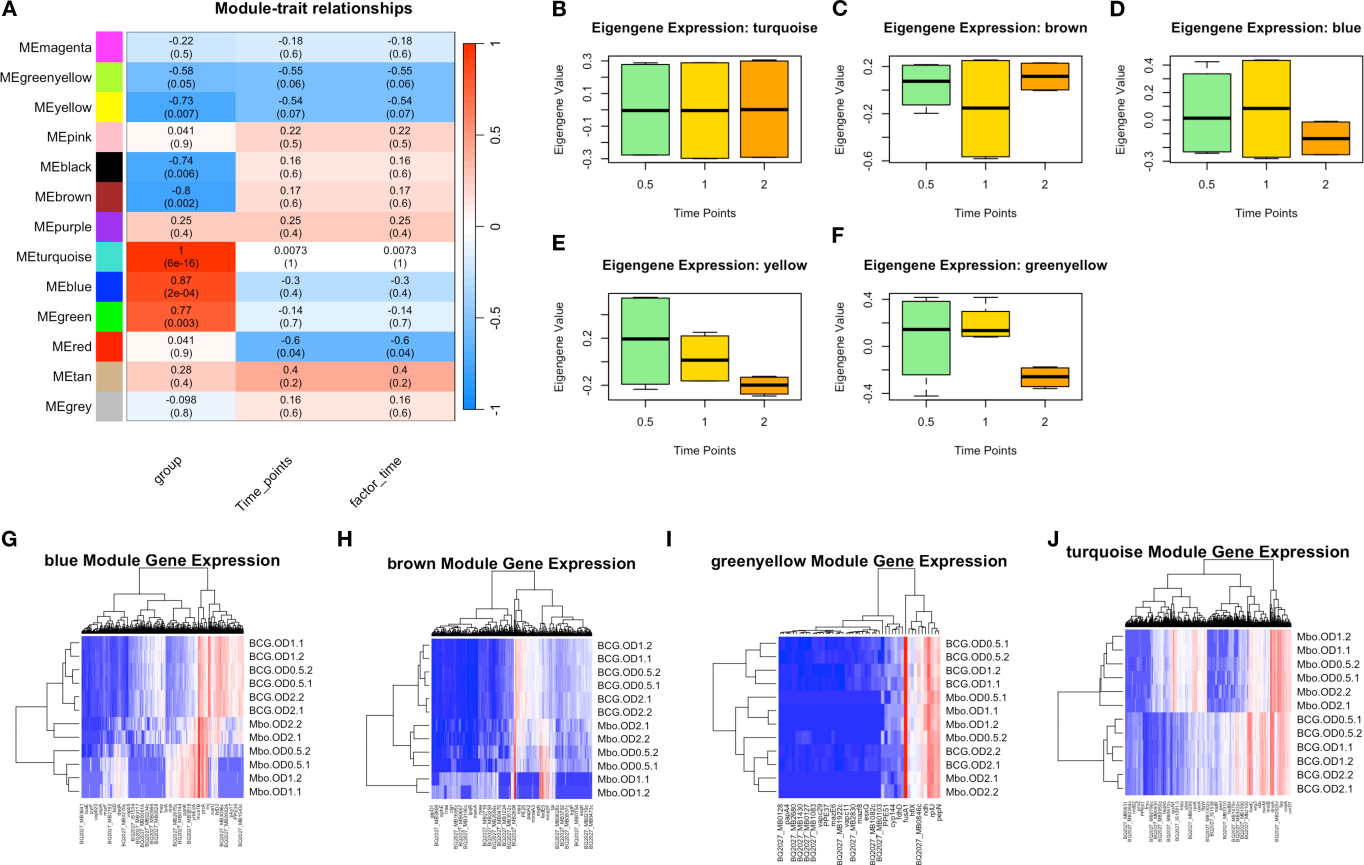
Key Weighted Gene Co-expression Network Analysis Identifies Modules Underlie Growth Phase and Strain Differences in *M. bovis* AF2122/97 and *M. bovis* BCG-Russia. **(A)** Module-trait relationships heatmap illustrating the correlation between WGCNA-identified gene modules and experimental traits, including bacterial strain, time points, and infection state. The heatmap shows the Pearson correlation values (R) and corresponding p-values (in parentheses) between module eigengenes and experimental conditions. The color scale indicates the strength and direction of correlation, with red representing positive correlation and blue representing negative correlation. **(B-F)** Boxplots of eigengene expression values for the turquoise, brown, blue, yellow, and green-yellow modules, respectively, across different time points, demonstrating dynamic module regulation over time. **(G-H)** Heatmaps of module-specific gene expression across different samples, with hierarchical clustering of genes and conditions: blue, brown, green-yellow, and turquoise modules. Red indicates high expression, while blue represents low expression, highlighting differentially regulated genes within each module.

To better characterize gene underlying the *M. bovis* adaptation at different growth phases, we employed Weighted Gene Co-expression Network Analysis (WGCNA) to identify co-expressed gene networks and their relationships with experimental traits (**Figure 3**). This analysis revealed 13 distinct gene modules (**Supplementary Table S2**, **Supplementary Figure 3**) that show correlation with bacterial growth phase progression, reinforcing the phase-specific regulatory mechanisms governing virulence and metabolic adaptation.

The module-trait correlation analysis (**Figure 3A**) revealed that five modules (turquoise, blue, brown, yellow, and greenyellow) exhibited significant correlations with bacterial growth phase progression. While not all modules exceeded an R-value of 0.6, their eigengenes expression (co-expressed genes based on WGCNA) trends (**Figure 3B**) aligned with observed DEGs and strain-specific transcriptomic behavior, prompting their inclusion in subsequent functional analyses.

The turquoise module I (1,626 genes) exhibited the strongest correlation with bacterial growth phase (r = 1.0, p < 0.01), suggesting its role in adaptive responses to metabolic shifts during transition from exponential to stationary phase. Genes within this module included several lipid metabolism regulators, such as *fadD21*, *fadD22*, *fadD23*, *fadD25*, *fadD26*, *fadD28*, *fadD29*, and *fadD30*, which are involved in fatty acid degradation and lipid utilization (**Figures 3A, B, J**), processes essential for energy metabolism and adaptation to stationary phase (Simeone et al., 2010). Additionally, the presence of *yrbE2A*, *fusA2b*, *rnj*, and *mmpL3* suggests that this module also contributes to membrane transport and stress adaptation (Su et al., 2021), enabling bacterial survival under nutrient-limited condition which is considered key factors for mycobacterial virulence during infection.

The blue module II, (695 genes), exhibited a strong negative correlation with the bacterial strain factor (r = -0.40, p < 0.05), indicating an inverse relationship—higher expression in M. bovis relative to BCG—which is a valid and interpretable outcome within the WGCNA framework (Langfelder and Horvath, 2008). This module was enriched with genes associated with virulence and host-pathogen interactions. Notably, *eccC2_2* and *mmsA*, key components of the ESX-1 secretion system and methylmalonate metabolism, respectively, were strongly upregulated in *M. bovis* compared to *M. bovis* BCG (**Figures 3A, D, G**) (Cole et al., 1998). The presence of PPE and PE_PGRS family genes further suggests that this module plays a crucial role in immune evasion and intracellular persistence, consistent with the virulent phenotype of *M. bovis* (Domenech et al., 2005).

Conversely, the brown module III, (466 genes), which exhibited a moderate correlation with growth phase (r = 0.35, p < 0.05), contained genes linked to central metabolism and oxidative stress response, including *scoA*, *BQ2027_MB1086*, and *fadE22a* (**Figures 3A, C, H**) (Bitter et al., 2009). The upregulation of these genes in *M. bovis* BCG suggests an enhanced reliance on metabolic pathways that compensate for the attenuation of virulence-associated factors (Gey van Pittius et al., 2006).

The yellow module IV, (193 genes), displayed a strong association with stationary phase adaptation (r = 0.73, *p* < 0.01) and contained genes such as *bfrB* and *desa2*, which are known to be involved in oxidative stress protection and lipid homeostasis (**Figures 3A, F**) (Geiman et al., 2006). The presence of these genes in this module suggests an essential role in long-term survival strategies, particularly for *M. bovis* BCG, which exhibits increased dormancy-associated gene expression (Brosch et al., 2007).

The green-yellow module V, (49 genes), was enriched with genes involved in cell wall remodeling and transport processes, such as *mce1D, espc, and yrbE2A*, which were significantly upregulated in *M. bovis* compared to BCG (**Figures 3A, F, I**) (Reddy et al., 2012). This module showed a moderate negative correlation with bacterial strain (r =-0.74, *p* = 0.03), indicating strain-specific expression differences rather than direct regulation by bacterial growth phase. The higher expression of these genes in *M. bovis* suggests a potential role in cellular integrity and nutrient uptake during early exponential growth. However, their expression declines over time, indicating that they are primarily utilized in the early stages of growth rather than throughout bacterial adaptation (Boon and Dick, 2002).

Overall, these findings provide a comprehensive view of the transcriptional programs governing *M. bovis* and *M. bovis* BCG physiology. Among the 13 identified co-expression modules, five were strongly associated with virulence, metabolic adaptation, and growth phase progression; turquoise, blue, brown, yellow, and green-yellow. These modules encapsulate key gene networks related to lipid metabolism, immune evasion, oxidative stress response, dormancy, and cell wall remodeling. Together, they highlight distinct strategies employed by the virulent and attenuated strains to adapt to environmental shifts and host-related pressures.

### Transcription factor enrichment analysis

**Figure 4:**
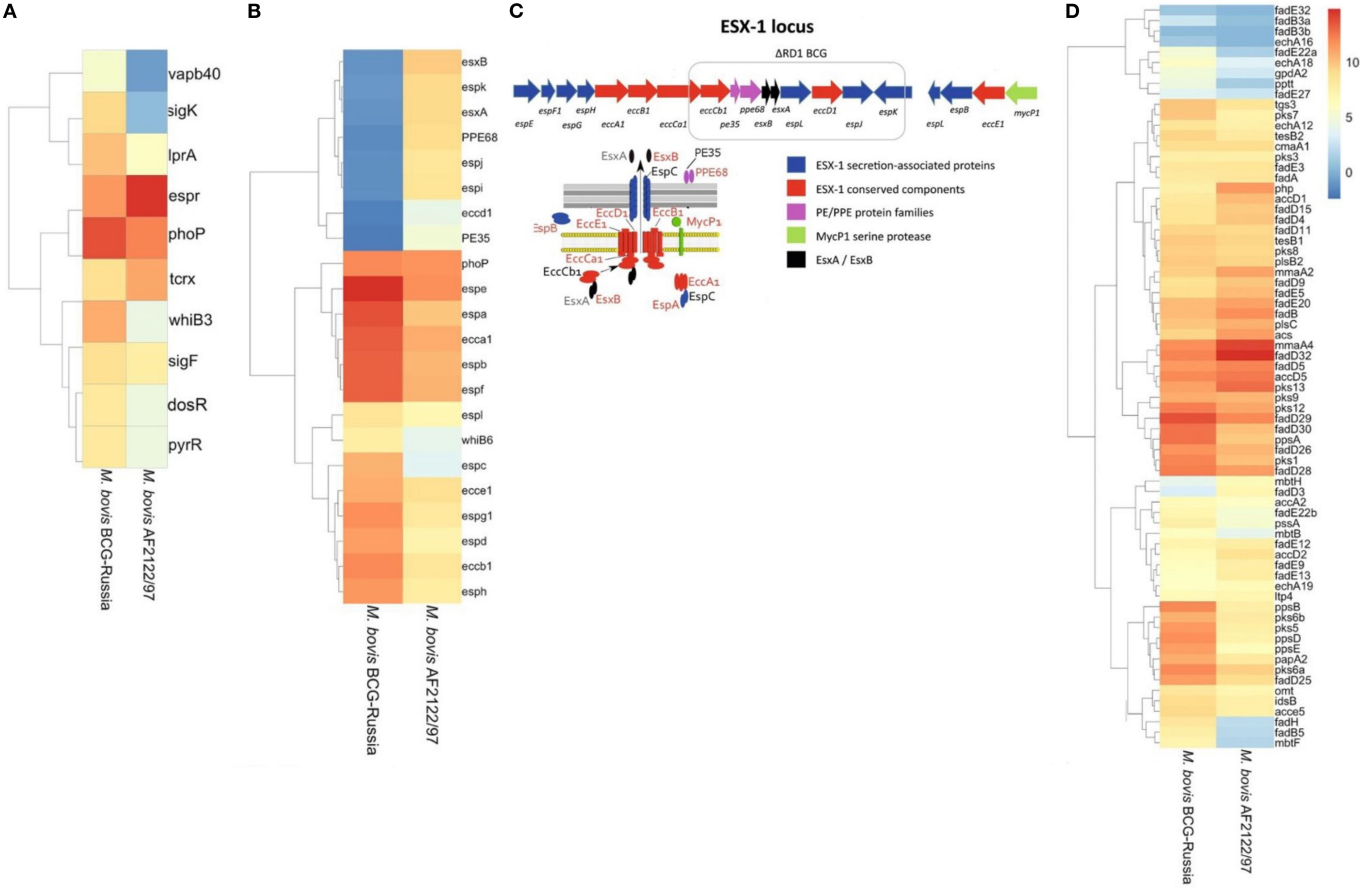
Differential Expression and Genomic Architecture of the ESX-1 Locus in *M. bovis* and BCG. Differentially expressed genes [|log_2_FC| > 1.5, FDR < 0.05] identified across all growth phases (OD600 = 0.5, 1.0, and 2.0) comparing M. bovis AF2122/97 and M. bovis BCG-Russia are shown. **(A)** Heat map displaying transcriptional factors and virulence-associated genes. **(B)** Heat map displaying ESX-1-associated genes, highlighting the deletion of the RD1 region in BCG and its impact on secretion system components. **(C)** Schematic representation of the ESX-1 locus, highlighting the RD1 deletion in BCG strains. Genes encoding ESX-1 secretion-associated proteins (blue), conserved ESX-1 components (red), PE/PPE family proteins (purple), and the MycP1 serine protease (green) are illustrated, along with interactions between secretion system components. **(D)** Heat map displaying genes involved in lipid metabolism and cell envelope biosynthesis. Note scale bar for the heat maps where numbers indicate log_2_ fold−change (*M. bovis* vs BCG): ≤ –1.5 (blue) to 0 (white) to ≥ +1.5 (red); zero-change baseline is white.

*M. bovis* encodes nearly 200 transcriptional factors, similar to *M. tuberculosis* (Smith, 2003). The differential gene expression observed between the virulent and the attenuated bovine tubercle bacilli may be a consequence of differences in global transcriptional regulators between the two species. To address this hypothesis, a curated transcription factor enrichment analysis was performed and revealed the significant association of 10 transcription factors (Turkarslan et al., 2015) (**Figure. 4A**). The transcription factors included in our analysis were selected based on known regulatory networks within the MTBC, and their differential expression (list fold of change used here) observed in our dataset and their known associations with virulence. This targeted approach allowed us to focus on the most relevant transcription factors impacting pathogenicity.

The association of transcription factors such as alternate sigma factors *SigK* and *SigF* along with cytoplasmic redox sensor *WhiB3* with the differentially expressed gene (DEG) lists indicates that disparate expression of virulence- relate pathways regulated by these transcription factors between the two pathogens could have significant consequence for infection (Gebhard et al., 2008; Veyrier et al., 2008). Importantly, *PhoP* and *EspR* were significantly differentially expressed, these transcription factors are important for adaptation of *M. bovis* to the intracellular environment and are functionally linked by such processes (Sherman et al., 2001; Singh et al., 2009). *PhoP* and *EspR* regulate the expression of ESX-1 secretion system-related genes. Furthermore, *PhoP* was expressed to a higher level in BCG; this may represent an attempt at a compensatory mechanism for aberrant PhoP signaling and supports previous reports of suboptimal *PhoP* signaling in *M. bovis* (Gonzalo Asensio et al., 2006; Walters et al., 2006; Gonzalo-Asensio et al., 2014). Additionally, 8 of 48 canonical *DosR* regulon, namely *BQ2027_MB1632c, BQ2027_MB1633c, BQ2027_MB1634c, BQ2027_MB1636c, BQ2027_MB1650, BQ2027_MB1653, BQ2027_MB2030*, and *BQ2027_MB2033* (Sivaramakrishnan and de Montellano, 2013; Belardinelli et al., 2025) were significantly upregulated in *M. bovis* virulent strain, consistent with the role of DosR in early adaptation to hypoxic or stress conditions. This differential expression pattern may reflect the integration of DosR responses with other transcriptional regulators such as *PhoP*, *EspR*, and *WhiB3* (Gonzalo Asensio et al., 2006; Gonzalo-Asensio et al., 2014). *DosR* regulon is important during conditions that do not allow aerobic respiration (Leistikow et al., 2010), like in the lungs of *M. tb*-infected mice (Voskuil et al., 2003) and in interferon-gamma-activated murine macrophages (Schnappinger et al., 2003).

Interestingly, several genes that were in the deleted regions from BCG were highly expressed in *M. bovis* (**Supplementary Figure 4**) including the major antigens ESAT-6 and CFP10, secreted by the ESX-1 secretion system of the MTBC, a system which has been implicated in mycobacterial escape from the phagosome to the cytosol that results in a Type-I interferon response within the infected macrophage (Simeone et al., 2009; Simeone et al., 2012; Simeone et al., 2015a). As *EspR* is induced in *M. bovis* AF2122/97, there is a significant induction of the ESX-1 *secretion system in M. bovis*, including *esx-1-*related proteins such as *esxA*, *espA*, *espC*, *espD* (Raghavan et al., 2008; Simeone et al., 2009; Chen et al., 2013; Solans et al., 2014; Cao et al., 2015) (**Figure 4B**). Additionally, all genes related to RD1 region (**Figures 4B, C**) was observed to be diminished expression in *M. bovis* BCG-Russia, this is an emphasis on the identity of each *strain* as RD1 is absent from the vaccinal strain. Alternate transcriptional regulation between the *M. bovis* BCG-Russia and the *M. bovis* AF2122/97 may represent differential priming events in preparation for the initial interactions of both species with their respective host immune systems. Increased expression of the ESX-1 secretion system may facilitate faster escape of *M. bovis* AF2122/97 from the phagosome into the cytosol in contrast to BCG, hence triggering DNA-sensing pathways and increased IFN response seen in our data (Simeone et al., 2012; Simeone et al., 2015b).

### Lipid metabolism enrichment analysis

The results of transcriptomic analysis indicated subtle difference in lipid metabolism related genes between the virulent and the attenuated bovine tubercle bacilli. *M. bovis* AF2122/97 showed higher expression of the *Pks13/FadD32* pair (**Figure 4D**), which are involved in the Biosynthesis of Mycolic Acids (Gavalda et al., 2009). FadD32 gene is adjacent to pks13, this genetic loci, *fadD32-pks13*, is conserved also in *M. tb* and *M. leprae* (Marrakchi et al., 2014), as this gene cluster is restricted to mycolic-acid-producing bacterial species (Gande et al., 2004). FadD32, a fatty acyl- AMP ligase, is involved in catalyzing the formation of acyl-adenylates, the activated form of meromycolic acid substrate in the mycolic condensation reaction (Trivedi et al., 2004). It also assists the transfer of the meromycoloyl chain onto the N-terminal acyl carrier protein (ACP) domain of the condensing enzyme Pks13 (Le et al., 2016). While Pks13 is a unique polyketide synthase (PKS) forms the a-alkyl β-ketoesters which is the direct precursors of mycolic acids (Gavalda et al., 2009). Importantly, Polyketide synthase Pks13 and its acyl−AMP ligase partner FadD32, encoded within the same locus, are universally regarded as essential for mycolic acid biosynthesis and bacterial viability in the *M. tb* complex (Portevin et al., 2004). Also, Pks13/FadD32 pair have been shown to be required for virulence in M. tb (Sassetti and Rubin, 2003; Mukhopadhyay et al., 2012). The *M. bovis* virulent strain orthologues may also, therefore, play a role in virulence. Upregulation of *Pks13/FadD32* pair (log_2_ fold change ranging from 2.3 to 4.3 across growth phases) may indicate divergent expression of Mycolic acid between the virulent and attenuated strains of *M. bovis*, an observation that can be reflected on the composition of the cell wall of each bacillus.

*MmaA4* was found also to be induced in the virulent *M. bovis*. *MmaA4*, a hydroxy- mycolate synthase, is also involved in mycolic acid modification by converting it to hydroxy mycolic acid, a precursor of methoxy‐ or keto‐mycolic acid (Alahari et al., 2009). It was found that MmaA4 modulates IL-12 production. *MmaA4* knockout mutant induced more IL-12 from murine macrophages and were attenuated for virulence in mice (Dao et al., 2008). As a result, the Δ*mmaA4* mutant strain induced significantly elevated levels of this critical Th1-type cytokine in macrophage cultures. Additionally, *MmaA4* knockout mutant of BCG vaccine induced higher levels of mycobacterial-specific multifunctional T cells, is more protective than BCG vaccine, and, surprisingly, may be safer than BCG when used in immunocompromised animals (Derrick et al., 2012; Derrick et al., 2016). Although BCG-Russia exhibited overall lower expression of certain lipid metabolic enzymes, the functional consequences of these differences require further investigation.

Interestingly, *BQ2027_Mb2982c*, encoding a glycosyltransferase involved in the synthesis of the trisaccharide phenolic glycolipid (PGL) that is derived from phthiocerol dimycocerosates (PDIM), was found to be expressed at higher levels in *M. bovis* BCG-Russia (data not shown). However, it should also be noted that *BQ2027_Mb2982c* is non-functional in *M. bovis*, so the higher expression of its gene may simply be due to loss of negative feedback inhibition. Previously, it was found that loss of PIDM/PGL reduces the protective efficacy of BCG vaccine (Tran et al., 2016). Since the loss of PDIM and PGL occurs naturally in a subset of BCG strains (Chen et al., 2007), it also suggests that these strains may have been over-attenuated, which compromises their effectiveness. Overall, most of these genes are participated in energy metabolism, including the fatty acid, cholesterol and glycolipid metabolism. It is postulated that pathogens downregulated its metabolic activity to reduce energy consumption and to persist in a prolonged dormant state.

### Validation of DEGs *in vivo*

**Figure 5:**
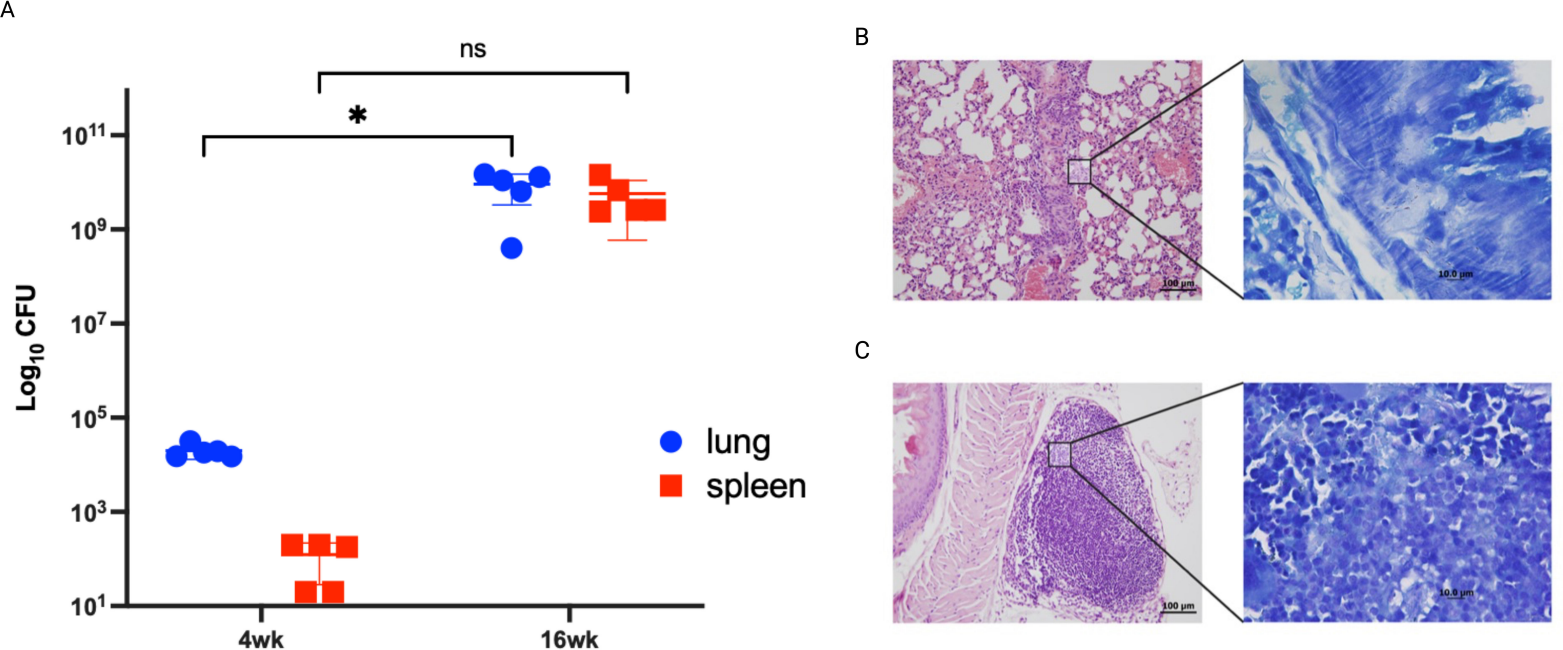
Infection confirmation through bacterial burden and histopathology. Groups of C3HeB/FeJ mice were infected by aerosol route with *M. bovis* strain AF2122/97. Lungs from infected animals were harvested and cultured at 4- and 16- weeks post infection. Each circle represents the colonization level for each organ from one animal. Asterisks (* for p < 0.05 and ** for p < 0.005) indicate statistically significant difference in colonization level between 4- and 16- weeks post infection **(A)**. Tissues sections stained with H&E collected from mice lungs infected with *M. bovis* AF2122/97 at 4 weeks post infection **(B)** and 16 weeks post infection **(C)**. **(B, C)** are shown at 40 × magnification (scale bar = 200 μm). Insets showing Ziehl-Neelsen- stained lung sections are also included, with arrowheads indicating acid-fast bacilli at 1000× magnification (scale bar = 100 μm). No bovine TB-associated granuloma infiltrates or acid-fast bacilli were found in any tissues in the naive group.

As we were able to identify *in vitro* differentially expressed genes between attenuated and virulent *M. bovis* strains. To validate the biological relevance of key genes identified *in vitro*, we analyzed gene expression of a limited set of genes in a susceptible murine model infected with the virulent strain. Lung tissues were analyzed using qRT-PCR, a sensitive assay for gene expression, to confirm their expression during *in vivo* infection. Our analysis focused on key *in vitro* expressed differentially expressed genes change their expression during the infection of the host tissue using the C3HeB/FeJ mouse model. We chose this model as it recapitulates the hallmark of bovine tuberculosis lung lesions following *M. bovis* aerosol infection (Boute et al., 2017). Mouse groups were sampled at 4- and 16- weeks post-infection to represent early and progressive stages of infection following aerosol infection with *M. bovis* AF2122/97. colonization levels increased at 4 w.p.i but peaked by 16 w.p.i (**Figure 5a**). Histologically, type I lesions resulted from the occlusion of alveolar spaces by a cellular infiltrate were noticeable by 4 w.p.i (**Figure 5b**). However, previous studies of *M. bovis* infection in C3HeB/FeJ murine model showed that type I lesions that evolved into an organized granuloma with a central accumulation of foamy macrophages were only visible by 5 w.p.i (Boute et al., 2017). This might explain the less organized lesion observed in the examined mouse lungs. As infection progressed, the inflammatory responses were intensified in lungs by 16 w.p.i with the observation of central necrosis in type I lesions (**Figure 5c**). These histological findings agree with the increase of bacterial burden by progression of infection.

**Figure 6:**
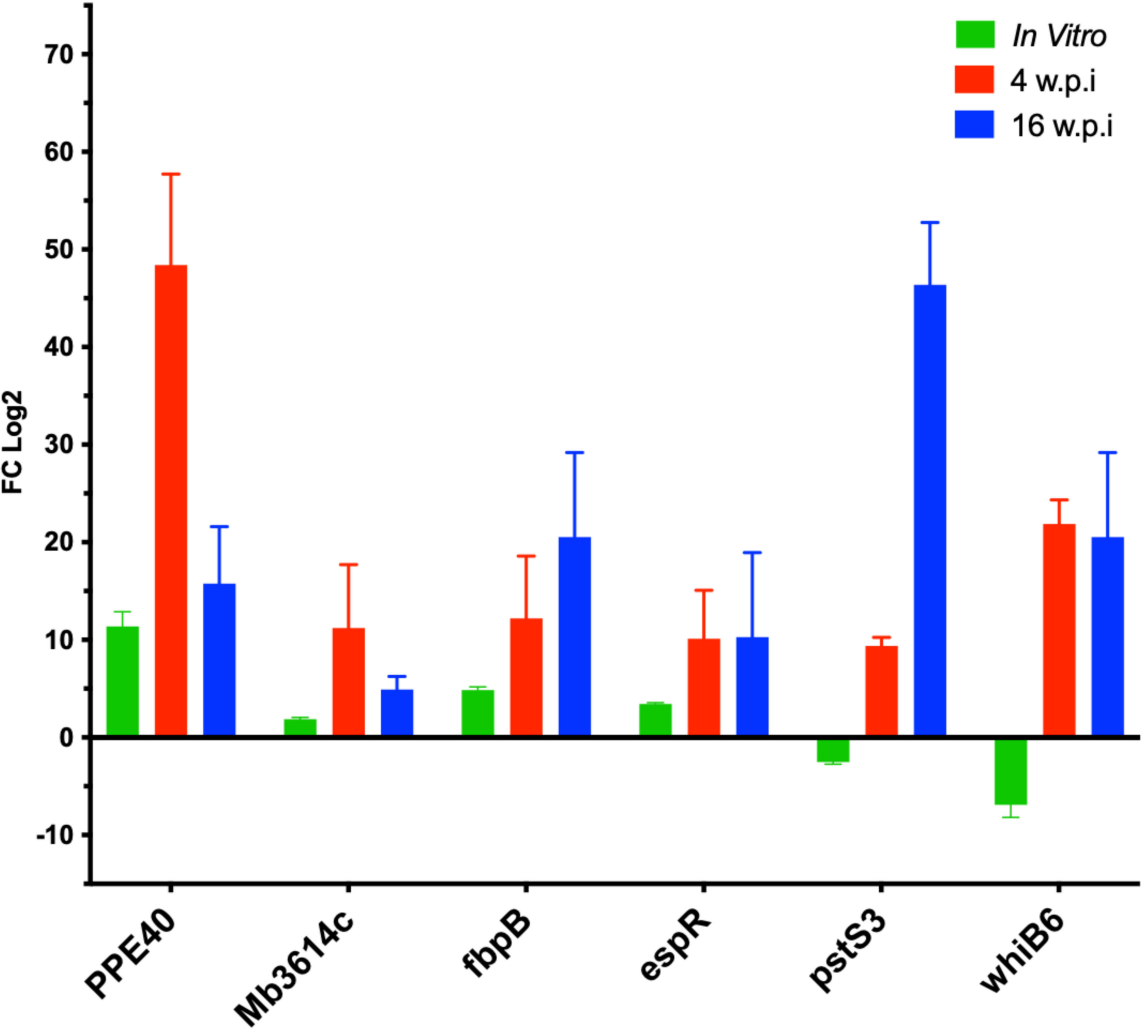
The relative expression of selected genes by qRT-PCR analysis. quantitative real- time PCR analysis of total RNA extracted from lung tissue samples collected C3HeB/FeJ mice groups at 4 w.p.i and 16 w.p.i. Expression levels were calculated with ΔΔCt relative quantitation method relative to the gene expression in the mid-log phase *In-Vitro* culture. At each time point, samples from 5 animals in each group were included and standard errors of the mean (SEM) of the three measurements were presented as error bars.

Several transcripts were selected to be quantified using quantitative RT-PCR (**Figure 6**), from bacterial RNA purified from the murine lung tissue based on their differential expression magnitude, known virulence associations, relevance to secretion or lipid metabolism pathways and their potential involvement in adaptation of *M. bovis* into host microenvironment a as suggested previously (Brosch et al., 2007; Malone et al., 2018). Among the highly regulated genes *Mb3614c* (*BQ2027_MB3614c*), *pstS3* (*BQ2027_MB0951*), *PPE40* (*BQ2027_MB2377c*), *fbpB* (*BQ2027_MB1918c*), *whiB6* (*BQ2027_MB3892c*) and *espR* (*BQ2027_MB3910c*). Interestingly, most of the genes whose expression dramatically changed between virulent and attenuated strains during *in vitro* culture showed similar differential expression during lung tissue infection (**Figure 5**). For example, Mb3614c is a putative transcription factor reported to play a regulatory role under starvation conditions (Ramos et al., 2020), was found to be also induced during early stage of lung tissue infection. Similarly, PPE40, a hypothetical PPE-family protein predicted to be an outer membrane protein, which is a part of the ESX 5 secretion system only found in pathogenic slow growing mycobacteria (Baena et al., 2019). It was found that PPE40 was induced more at early phase of infection. While *pstS3* (*BQ2027_MB0951*) a periplasmic phosphate-binding lipoprotein (Garnier et al., 2003) was found to be induced to more extent at earlier stages of infection, while downregulated in *In-Vitro* culture. The pstS3 is a known as a component of this primary phosphate uptake system and reported previously to be highly expressed in mouse lungs (Shi et al., 2004). Moreover, PstS3 is an excellent immunogen inducing CD8+ T-cell activation and both Th1 and Th17 immunity (Palma et al., 2011). Also, mice vaccinated with DNA coding for pstS3 demonstrated significant and sustained reduction in bacterial load in lungs after *M. tb* challenge (Tanghe et al., 1999). The observation of *pstS3* induction during lung infection while downregulated during *In-Vitro* growth highlights the differential expression of virulence factors specific for the adaptation of *M. bovis* to host microenvironment.

## Conclusions

This study provides a comprehensive analysis of the transcriptional differences between the virulent *M. bovis* AF2122/97 and the attenuated *M. bovis* BCG-Russia strains. Through RNAseq, we identified significant differentially expressed genes (DEGs) associated with various growth phases and their roles in virulence and survival. Our findings highlight the pronounced upregulation of virulence-associated genes such as esxA and phoP in the virulent strain, contrasting with the stress response-related gene sigH in BCG. The comparison across different growth phases revealed that *M. bovis* adapts its metabolic and virulence strategies according to the growth phase. Notably, genes like *icl1* and *mbtH* were upregulated in the virulent strain at mid-log, underscoring their roles in lipid metabolism and iron acquisition, respectively. At stationary phase, the significant upregulation of dormancy-associated genes *dosR* and *relA* in *M. bovis* indicates its preparedness for long-term survival under adverse conditions, a stark contrast to the elevated *groEL* expression in BCG, which suggests a focus on stress response. The transcription factor enrichment analysis identified key regulators, including *sigK*, *sigF*, *phoP*, and *espR*, which play crucial roles in the differential expression of virulence-related pathways. These transcription factors underscore the complex regulatory networks that drive the pathogenicity of *M. bovis*. It is important to note that BCG sub-strains vary substantially in terms of genomic deletions, antigen expression, and immunogenicity. As our findings are derived from BCG Russia, an early sub-strain, the observed transcriptomic patterns may not fully reflect those of later sub-strains. This consideration is essential when interpreting generalizability of BCG-associated responses. Additionally, the study identified critical differences in lipid metabolism genes, such as the *Pks13/FadD32* pair and *MmaA4*, further linking these pathways to the virulence and survival strategies of the pathogen. The findings from the *in vivo* experiments using the C3HeB/FeJ mouse model corroborated the *in vitro* data, particularly the role of *Mb3614c*, PPE40, and *pstS3* in adapting to the host environment.

Overall, this work provides valuable insights into the molecular mechanisms underlying the virulence and attenuation of *M. bovis*. The identified DEGs and transcription factors present promising targets for future research aimed at developing novel therapeutic strategies and improving tuberculosis control measures. Further studies on these candidate genes could elucidate their roles in the survival strategies of *M. bovis* within host tissues, paving the way for more effective interventions against bovine tuberculosis.

## Data Availability

The data presented in the study are deposited in the GEO repository, accession numbers: BCG-1_1: GSM9241655, BCG-1_2: GSM9241656, BCG-2_1: GSM9241657, BCG-2_2: GSM9241658, BCG-0.5_1: GSM9241659, BCG-0.5_2: GSM9241660, Mbovis-1_1: GSM9241661, Mbovis-1_2: GSM9241662, Mbovis-2_1: GSM9241663, Mbovis-2_2: GSM9241664, Mbovis-0.5_1: GSM9241665, Mbovis-0.5_2: GSM9241666.

## Supplementary material

Supplementary Table 1 Primers’ sequences used in this study.

Supplementary Table 2 Summary of WGCNA gene co-expression modules and their correlations with experimental traits. This table lists each identified module along with its correlation values to group (strain), time points, and factor_time (growth phase), associated p-values, and the total number of genes per module. Modules were detected using dynamic tree cutting following TOM-based hierarchical clustering. Correlation values (r) represent Pearson correlation between each module eigengene and experimental traits. P-values were computed using Student asymptotic test (WGCNA corPvalueStudent). Only modules with |r| > 0.5 and p < 0.05 were considered biologically meaningful in downstream analyses. Modules with lower or nonsignificant correlations are retained for completeness but not emphasized in the main text.

Supplementary Figure 1 Growth kinetics of *M. bovis* AF2122/97 and *M. bovis* BCG Russia *In-Vitro* cultures. **(A)** The number of colony forming units (‘CFU/ml’) measured at specific OD600 measures representing log, stationery and lag phases. **(B)** OD600 measurement over time in days. The dashed line indicates the limit of detection. CFU counts were determined by culturing on 7H10 Middlebrook media with serial dilutions. Shown are one of two similar biological replicates with error bars representing standard deviation.

Supplementary Figure 2 Overview of RNAseq quality. **(A)** Pearson correlation distance matrix of reads mapped to *M. bovis* genes in the six *M. bovis* AF2122/97 and six *M. bovis* BCG Russia RNA-seq datasets. **(B)** The sequencing library size bar plot showing many reads we have for each sample, dashed line indicating cutoff for library coverage.

Supplementary Figure 3 Weighted Gene Co-Expression Network Analysis (WGCNA) module identification and network topology assessment. **(A)** Hierarchical clustering dendrogram of genes based on topological overlap, with branches representing gene clusters and module assignments indicated by different colors. The Dynamic Tree Cut method was applied to define modules. **(B)** Scale-free topology model fit (signed R²) as a function of the soft-thresholding power. A power of X was chosen as it reached the threshold of R² > 0.9, ensuring approximate scale-free topology. **(C)** Mean connectivity of the network as a function of the soft-thresholding power. Connectivity decreases as the power increases, supporting the selected threshold for a biologically meaningful co-expression network. This analysis enables the identification of co-expressed gene modules and their relevance to bacterial growth phase and virulence-associated transcriptional programs.

Supplementary Figure 4 Differential expression of RD region genes in *M. bovis* compared to *M. bovis* BCG during early logarithmic growth. Barplot displaying log_2_ fold change values for genes located within the canonical Region of Difference (RD) loci that are deleted in BCG but present and expressed in virulent *M. bovis* AF2122/97. Gene expression was compared between *M. bovis* at OD600 = 0.5 (early log phase). Positive values indicate upregulation in *M. bovis*, and negative values represent downregulation. These genes include several ESX-1 components (e.g., eccC1, espA) and PE/PPE family members implicated in virulence and host-pathogen interactions. This standalone summary highlights the expression profile of RD genes distinguishing virulent from attenuated strains.
