# Supplementary material for: Comparative transcriptomic signatures of virulent and attenuated Mycobacterium bovis growing in vitro and in mice
Source: Front Cell Infect Microbiol. 2025 Oct 28;15:1643664. doi: 10.3389/fcimb.2025.1643664 (PMC12604023; doi:10.3389/fcimb.2025.1643664)
Supplement: Supplementary Table 1 — Primers’ sequences used in this study. [file Table1.docx]

**Supplementary Table 1.** Primers’ sequences used in this study

| PPE40_F | TTGCAAACATTGGCTCTTTC |
| --- | --- |
| PPE40_R | TCTGGCTACCCGAATTTACC |
| Mb3614c_F | GAAGGCCTGGACAAGGTTT |
| Mb3614c_R | GATGCGAGTTTCTCGAGGTT |
| fbpB-f | CCTGCGGTTTATCTGCTCGA |
| fbpB-r | TGTAGAAGCTGGACTGCCCG |
| espR-f | AGATGGACCCCCAAGTCG |
| espR-r | GGACCAACTTCCTTCAGCAG |
| pstS3_F | CTCGACACGGACTCGTTCTA |
| pstS3_R | GCAAACGATTTCGTATGTCG |
| whiB6-f | CATGACAGTAACCGCCCTGT |
| whiB6-r | TTCGGGAATTACGACCCCTG |
