# Supplementary material for: Comparative transcriptomic signatures of virulent and attenuated Mycobacterium bovis growing in vitro and in mice
Source: Front Cell Infect Microbiol. 2025 Oct 28;15:1643664. doi: 10.3389/fcimb.2025.1643664 (PMC12604023; doi:10.3389/fcimb.2025.1643664)
Supplement: Supplementary Table 2 — Summary of WGCNA gene co-expression modules and their correlations with experimental traits. This table lists each identified module along with its correlation values to group (strain), time points, and factor_time (growth phase), associated p-values, and the total number of genes per module. Modules were detected using dynamic tree cutting following TOM-based hierarchical clustering. Correlation values (r) represent Pearson correlation between each module eigengene and experimental traits. P-values were computed using Student asymptotic test (WGCNA corPvalueStudent). Only modules with |r| > 0.5 and p < 0.05 were considered biologically meaningful in downstream analyses. Modules with lower or nonsignificant correlations are retained for completeness but not emphasized in the main text. [file Table2.docx]

**Supplementary Table 2: Summary of WGCNA gene co-expression modules and their correlations with experimental traits.** This table lists each identified module along with its correlation values to group (strain), time points, and factor_time (growth phase), associated p-values, and the total number of genes per module. Modules were detected using dynamic tree cutting following TOM-based hierarchical clustering. Correlation values (r) represent Pearson correlation between each module eigengene and experimental traits. P-values were computed using Student asymptotic test (WGCNA corPvalueStudent). Only modules with |r| > 0.5 and p < 0.05 were considered biologically meaningful in downstream analyses. Modules with lower or nonsignificant correlations are retained for completeness but not emphasized in the main text.

| Module | Group_correlation | Time_points | factor_time | P_value | Number_Of_Genes | Main_Function |
| --- | --- | --- | --- | --- | --- | --- |
| black | -0.22 | -0.18 | -0.18 | 0.57 | 98 | Protein folding and oxidative stress |
| blue | -0.58 | -0.55 | -0.55 | 0.063 | 695 | Virulence and ESX-1 secretion |
| brown | -0.73 | -0.54 | -0.54 | 0.071 | 466 | Central metabolism and respiration |
| green | 0.041 | 0.22 | 0.22 | 0.49 | 174 | Translation and ribosome biogenesis |
| greenyellow | -0.74 | 0.16 | 0.16 | 0.63 | 49 | Cell wall remodeling & transport |
| grey | -0.8 | 0.17 | 0.17 | 0.59 | 32 | unassigned / low connectivity gene |
| magenta | 0.25 | 0.25 | 0.25 | 0.43 | 62 | Hypothetical proteins & unknown function |
| pink | 1 | 0.0073 | 0.0073 | 0.98 | 79 | Small molecule transport |
| purple | 0.87 | -0.3 | -0.3 | 0.35 | 55 | Amino acid biosynthesis |
| red | 0.77 | -0.14 | -0.14 | 0.67 | 140 | PE/PPE genes and immune evasion |
| tan | 0.041 | -0.6 | -0.6 | 0.038 | 47 | Lipid synthesis |
| turquoise | 0.28 | 0.4 | 0.4 | 0.2 | 1650 | Lipid metabolism and stationary phase adaptation |
| yellow | -0.098 | 0.16 | 0.16 | 0.63 | 193 | \| Iron storage and dormancy \| \| --- \| |
