# Supplementary figures and images for: Comparative transcriptomic signatures of virulent and attenuated Mycobacterium bovis growing in vitro and in mice
Source: Front Cell Infect Microbiol. 2025 Oct 28;15:1643664. doi: 10.3389/fcimb.2025.1643664 (PMC12604023; doi:10.3389/fcimb.2025.1643664)

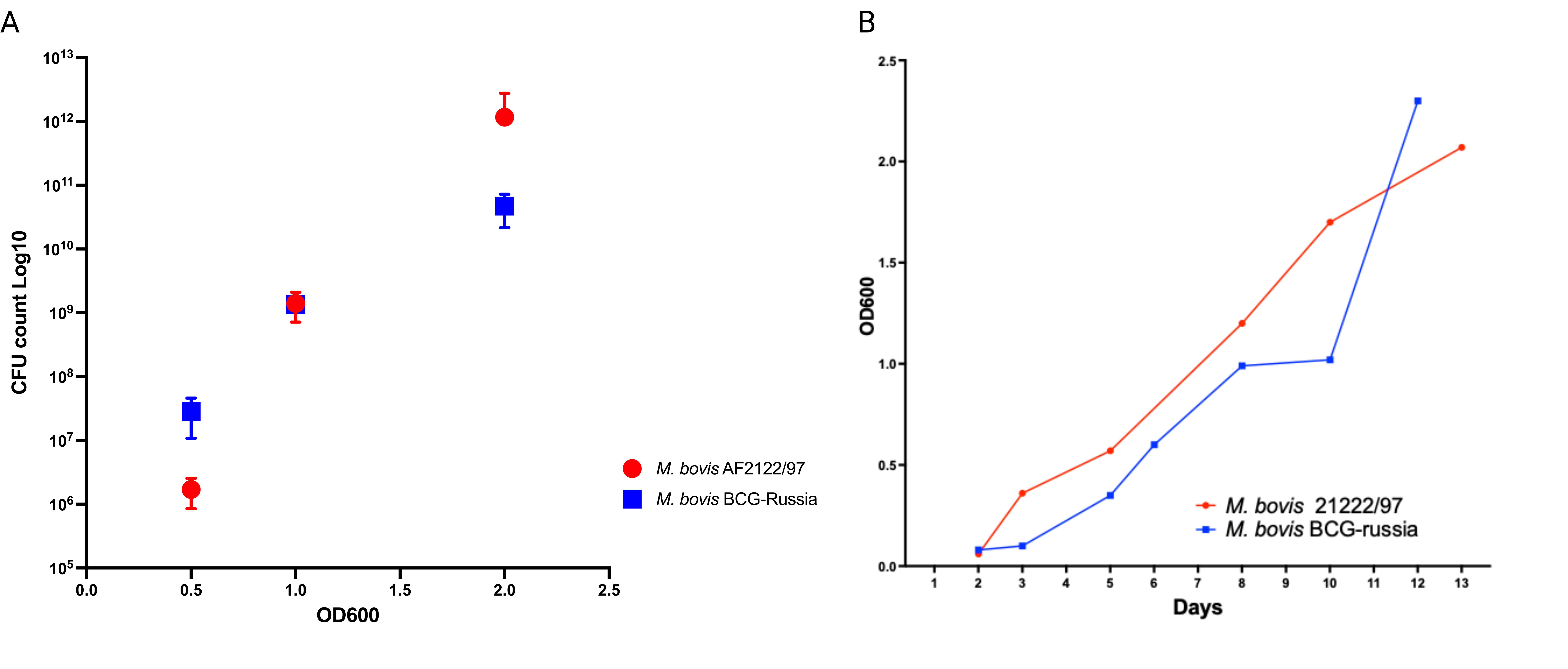

Supplement: Supplementary Figure 1 — Growth kinetics of M. bovis AF2122/97 and M. bovis BCG Russia In-Vitro cultures. (A) The number of colony forming units (‘CFU/ml’) measured at specific OD600 measures representing log, stationery and lag phases. (B) OD600 measurement over time in days. The dashed line indicates the limit of detection. CFU counts were determined by culturing on 7H10 Middlebrook media with serial dilutions. Shown are one of two similar biological replicates with error bars representing standard deviation. [file Image1.jpeg]

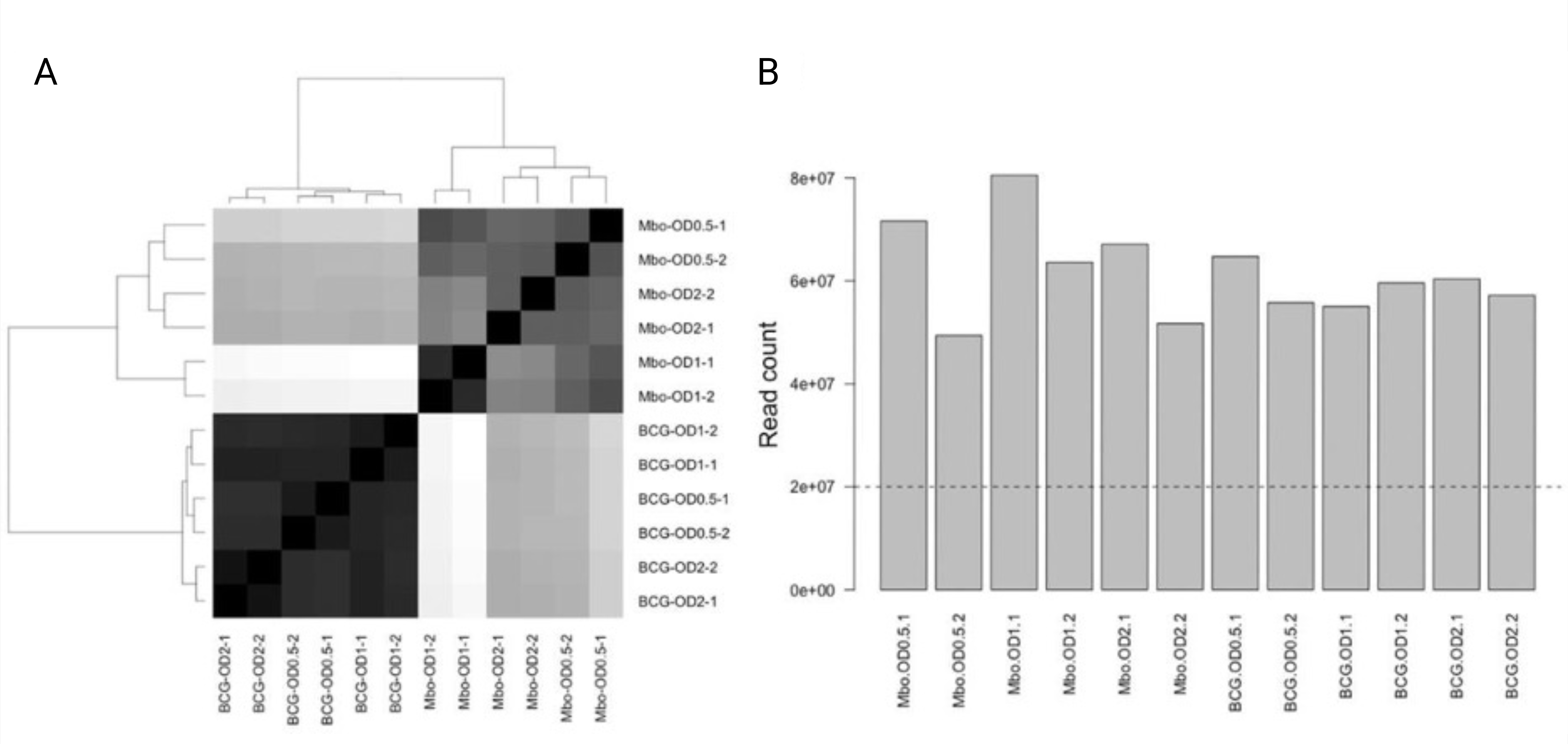

Supplement: Supplementary Figure 2 — Overview of RNAseq quality. (A) Pearson correlation distance matrix of reads mapped to M. bovis genes in the six M. bovis AF2122/97 and six M. bovis BCG Russia RNA-seq datasets. (B) The sequencing library size bar plot showing many reads we have for each sample, dashed line indicating cutoff for library coverage. [file Image2.jpeg]

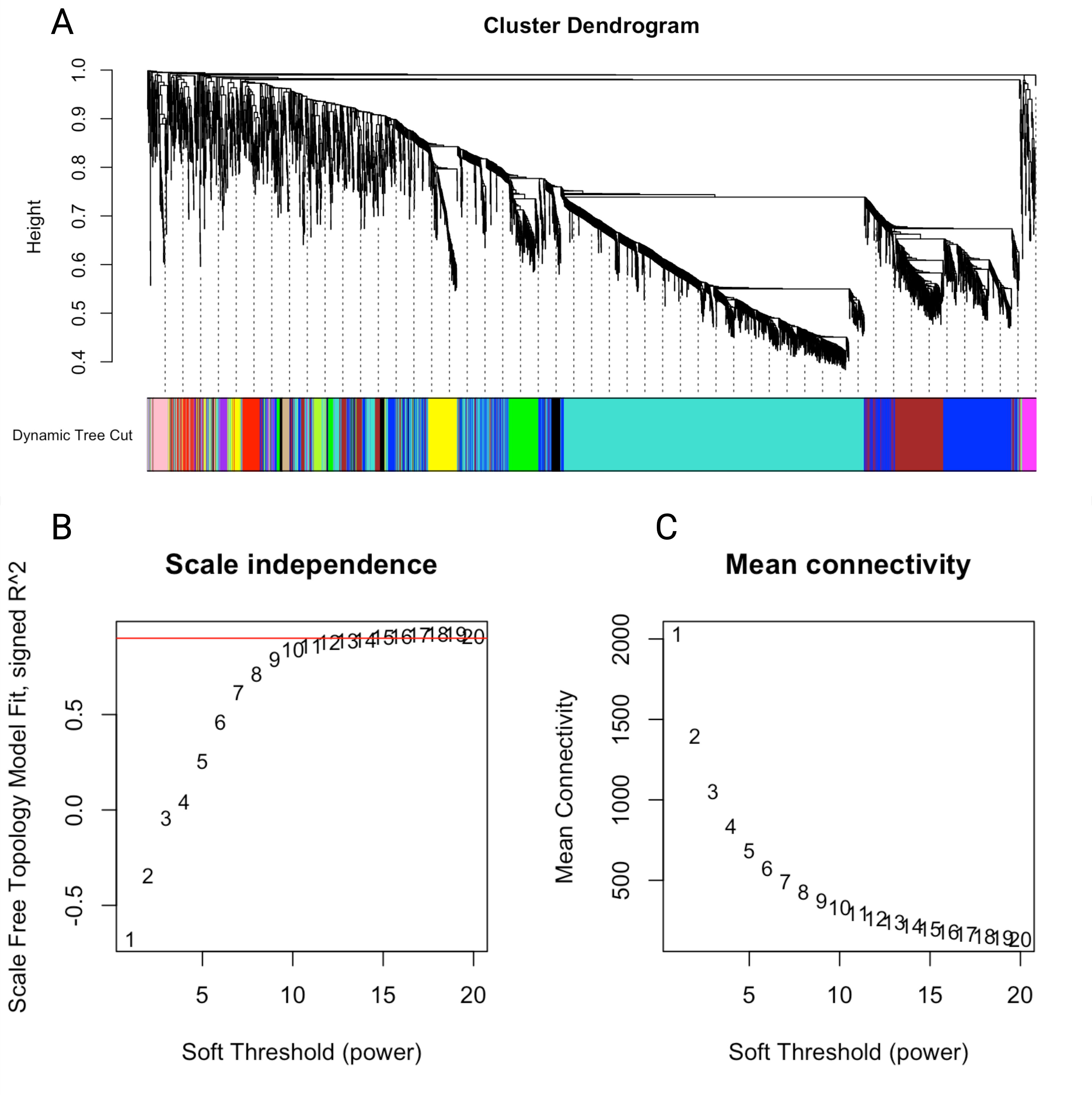

Supplement: Supplementary Figure 3 — Weighted Gene Co-Expression Network Analysis (WGCNA) module identification and network topology assessment. (A) Hierarchical clustering dendrogram of genes based on topological overlap, with branches representing gene clusters and module assignments indicated by different colors. The Dynamic Tree Cut method was applied to define modules. (B) Scale-free topology model fit (signed R²) as a function of the soft-thresholding power. A power of X was chosen as it reached the threshold of R² > 0.9, ensuring approximate scale-free topology. (C) Mean connectivity of the network as a function of the soft-thresholding power. Connectivity decreases as the power increases, supporting the selected threshold for a biologically meaningful co-expression network. This analysis enables the identification of co-expressed gene modules and their relevance to bacterial growth phase and virulence-associated transcriptional programs. [file Image3.jpeg]

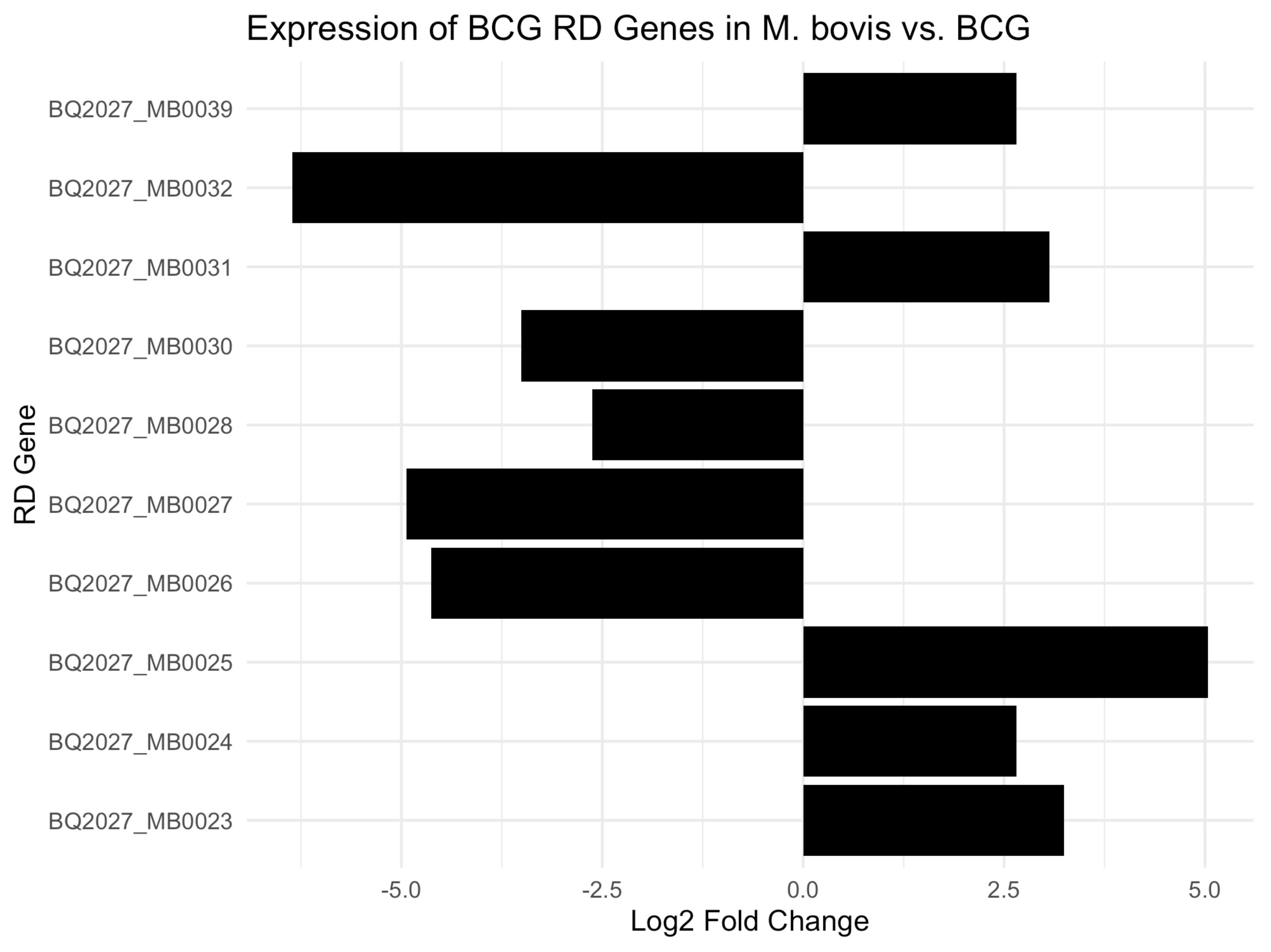

Supplement: Supplementary Figure 4 — Differential expression of RD region genes in M. bovis compared to M. bovis BCG during early logarithmic growth. Barplot displaying log2 fold change values for genes located within the canonical Region of Difference (RD) loci that are deleted in BCG but present and expressed in virulent M. bovis AF2122/97. Gene expression was compared between M. bovis at OD600 = 0.5 (early log phase). Positive values indicate upregulation in M. bovis, and negative values represent downregulation. These genes include several ESX-1 components (e.g., eccC1, espA) and PE/PPE family members implicated in virulence and host-pathogen interactions. This standalone summary highlights the expression profile of RD genes distinguishing virulent from attenuated strains. [file Image4.jpeg]
